# Reappraisal of the systematics of *Microglanis cottoides* (Siluriformes, Pseudopimelodidae), a catfish from southern Brazil

**DOI:** 10.1371/journal.pone.0199963

**Published:** 2018-07-05

**Authors:** Lenice Souza-Shibatta, João F. R. Tonini, Vitor P. Abrahão, Lucas R. Jarduli, Claudio Oliveira, Luiz R. Malabarba, Silvia H. Sofia, Oscar A. Shibatta

**Affiliations:** 1 Laboratório de Genética e Ecologia Animal, Departamento de Biologia Geral, Universidade Estadual de Londrina, Londrina, PR, Brazil; 2 Department of Organismic and Evolutionary Biology, Museum of Comparative Zoology, Harvard University, Cambridge, MA, United States of America; 3 Universidade de São Paulo, Museu de Zoologia da USP, São Paulo, SP, Brasil; 4 Faculdades Integradas de Ourinhos, Ourinhos, SP, Brasil; 5 Laboratório de Biologia e Genética de Peixes, Departamento de Morfologia, Instituto de Biociências, Universidade Estadual Paulista -UNESP, Botucatu, SP, Brazil; 6 Departamento de Zoologia, Universidade Federal do Rio Grande do Sul, Porto Alegre, RS, Brazil; 7 Departamento de Biologia Animal e Vegetal, Universidade Estadual de Londrina, Londrina, PR, Brazil; National Cheng Kung University, TAIWAN

## Abstract

The southern region of Brazil is characterized by high species diversity and endemism of freshwater fishes distributed across geographically isolated river basins. *Microglanis cottoides* has a widespread range across these river basins and occurs in sympatry with other endemic species of the genus (e.g. *M*. *cibelae*, *M*. *eurystoma*, and *M*. *malabarbai*). Herein we tested the monophyly of *M*. *cottoides* and presented for the first time information about the molecular phylogeny of species in the genus. The results suggest that *M*. *cottoides* currently forms a non-monophyletic group which includes populations endemic to the Uruguay River basin that are more closely related to *M*. *malabarbai*, and excludes *M*. *cibelae*, found to be nested within *M*. *cottoides*. Based on an integrative approach using morphological and molecular data, we propose *M*. *cibelae* as a junior synonym of *M*. *cottoides*, and the populations of the Uruguay River basin previously assigned to *M*. *cottoides* in fact belong to *M*. *malabarbai*. Our molecular phylogeny shows that *M*. *cottoides* is sister to *M*. *parahybae*, which is also a coastal species, and *M*. *malabarbai* is sister of *M*. *garavelloi*, both endemic to inland river basins. The time-calibrated phylogeny indicates that the separation between inland and the coastal clades occurred in the Tertiary period, and that the species within the coastal basins diverged in the Pliocene, which overlaps with the diversification times estimated for the two inland species as well. This pattern of diversification corroborates some previous studies with other fishes from the same region.

## Introduction

In southern South America, between 6 and 10 Kya, sea level oscillations in the Atlantic formed a series of coastal watersheds geographically isolated from each other, which drain directly into the ocean [1], [2]. In addition, this region comprises the Uruguay River basin, an inland drainage isolated from the coastal ones by the steep mountains on the eastern shore of the Brazilian crystalline shield [1]. As results of geomorphological and sea level variations, studies have identified smaller ecoregions [3] with high levels of endemism across coastal [4], [5], [6], [7] and inland drainages [8], [9], [10], [11] in southern South America.

*Microglanis* Eigenmann, 1912 (Pseudopimelodidae) are a group of fishes with small body size (standard length of less than 11 cm [12]) comprising 28 species distributed across South America [13], [14], [15], [16]. The geographic distribution of these fishes is affected by physio-ecological constraints since *Microglanis* do not tolerate salt water, which prevent species from dispersing through the sea, *sensu* Myers [17]. Four species of *Microglanis* occur throughout southern South America: *M*. *malabarbai* Bertaco and Cardoso 2005 and *M*. *eurystoma* Malabarba and Mahler-Jr., 1998 described from the Uruguay river basin, *M*. *cibelae* Malabarba and Mahler-Jr., 1998 described from the Tramandaí and Mampituba river basins in the coastal region of Rio Grande do Sul and Santa Catarina states, and *M*. *cottoides* (Boulenger 1891) described from the Laguna dos Patos drainage.

The taxonomic history of *Microglanis cottoides* is controversial among different authors. For instance, Gomes [18] considered *M*. *parahybae* (Steindachner 1880) as a junior synonym of *M*. *cottoides* due to the great morphological similarity. Mees [19], following the principle of priority, considered *M*. *parahybae* a senior synonym of *M*. *cottoides*. In contrast, Malabarba & Mahler-Jr [9] reviewed the taxonomy of *Microglanis* species in southern South America and considered both *M*. *cottoides* and *M*. *parahybae* as valid species. Thus, it was proposed that *M*. *cottoides* would occur in the Laguna dos Patos and Uruguay River drainages [9], [16], [20], [21], and that *M*. *parahybae* would be restricted to the Paraíba do Sul River basin. In addition, Malabarba & Mahler-Jr [9] described a new species from the southern South America (*M*. *cibelae*) geographically distributed in the Tramandaí (TRA) and Mampituba (MAM) river basins. Although *M*. *cottoides* has recently been listed in checklists and fish guides of Southern Atlantic river drainages [22], [23], [24], *Microglanis* populations in southern South America have not yet been revised on the basis of genetics and morphological data using a statistical framework to test whether current taxonomic decisions would receive support.

Identifications and descriptions of species solely based on morphological characters may present limitations when the characters used are masked by the effect of phenotypic plasticity and/or genetic variability [25]. This scenario would bring instability to species recognition and produce biased reports of species distribution and diversity. For this reason, in recent decades, several molecular tools have been used collectively with morphological data to study biodiversity. For instance, molecular tools have contributed to understanding the ontogenetic pattern of melanin in the lateral region of cyprinid fishes and led the authors to synonymize 12 nominal species [26].

The modern integrative taxonomy was formally introduced in 2005 to increase accuracy and reliability to delimit and describe taxa by integrating information from different kind of data and methodologies, such as molecular markers, ecological and morphological characters [27], [28]. In the last years, the increase of articles using multiple lines of evidence corroborates the success of the modern integrative approach, indicating a clear renewal of the taxonomy [29].

DNA barcoding is among the molecular tools applied in biodiversity studies, identification of cryptic species, and taxonomy [30], [31]. This technique has been quite effective for a wide variety of different taxa (amphibians—[32]; birds—[33]; bats—[34]; reptiles–[35], [36], and freshwater fish from the Neotropical region). This tool has been used with great success, in most cases identifying the species correctly [37], [38], [39], [40], [41], [42], [43], [44], or at different stages of the life cycle [45]. However, there are some criticisms regarding the use of this single fragment of mitochondrial DNA for the identification and description of new species, as well as for phylogenetic analyses [46], [47]. Nonetheless, several studies have shown that the DNA barcoding method is a valuable tool for taxonomy and systematics [25], [30], [48], [49].

Vogler et al. [50] argue against the usefulness of DNA barcoding in the delimitation and identification of species/lineages based only on genetic distances, since it does not take into account the differences in the time of divergence between them. However, improved statistical methods were proposed to delimit species with DNA barcoding data, such as the General Mixed Yule Coalescent Method (GMYC) [51], which is based on an evolutionary model incorporating the topology of a tree, which allows researches to differentiate between the interspecific (''diversification'') and intraspecific (''coalescence'') processes of the branching processes of the lineages [51], [52].

Here, we tested whether 1) *Microglanis cottoides* may comprise cryptic species across coastal drainages given the high level of endemism of other fish species; 2) endemic species of *Microglanis* in the coastal drainages from São Paulo to Rio Grande do Sul are synonyms of *M*. *cottoides*; and 3) populations from the Uruguay River basin belong to *M*. *cottoides*.

## Material and methods

### Ethical statement

All specimens used were collected in accordance with Brazilian laws, and the sampling was approved by the Sistema de Autorização e Informação em Biodiversidade (SISBIO number 12120–1) of the Instituto Chico Mendes de Conservação da Biodiversidade (ICMBio). After collection, the animals were anesthetized and sacrificed using 1% eugenol as approved by the Universidade Estadual de Londrina/UEL Ethics Committee on the Use of Animals (CEUA; protocol 37917–11) and accepted by the National Council for the Control of Animal Experimentation and Federal Board of Veterinary Medicine. The animals were preserved in 92.5^o^ GL ethanol and catalogued in the collection of the Museu de Zoologia da Universidade Estadual de Londrina (MZUEL), Londrina, Paraná, Brazil.

### Molecular analyses

#### Taxon sampling, extraction and sequencing

**Fig 1:**
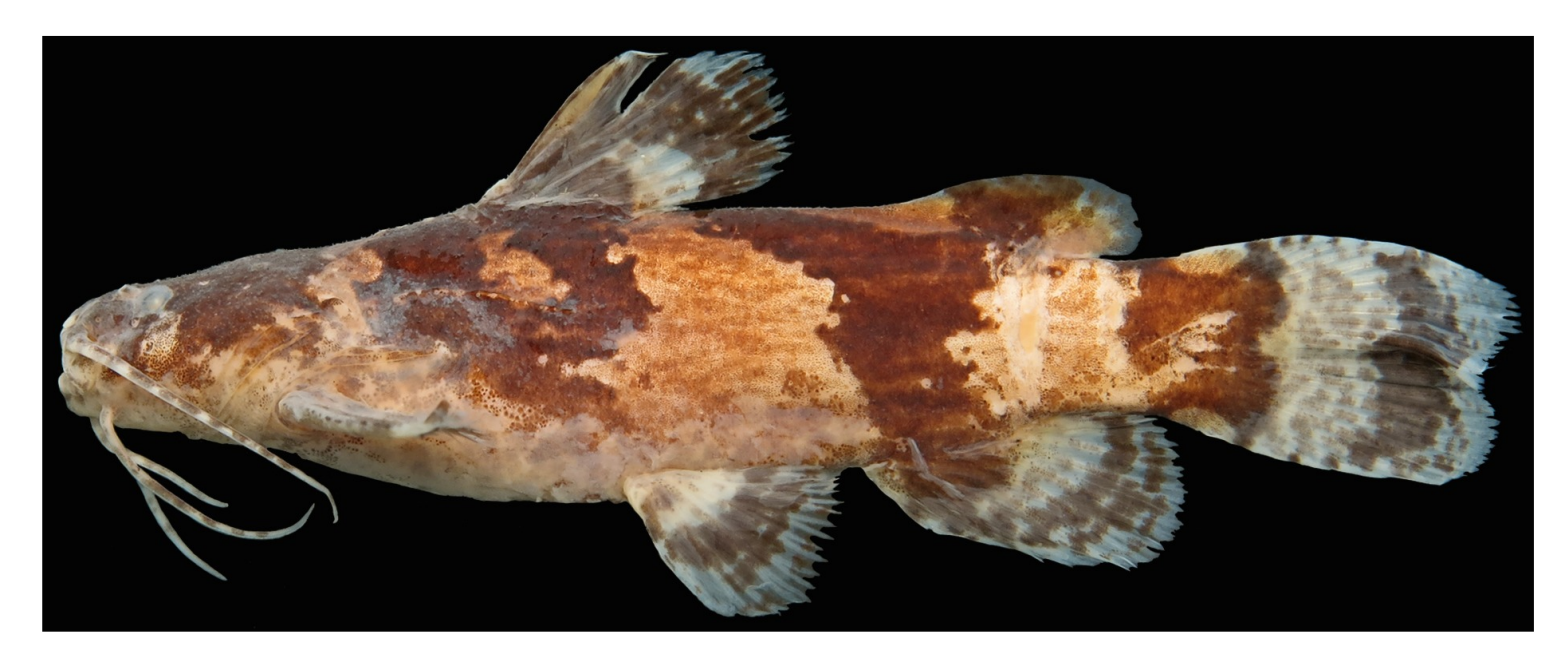
*Microglanis cottoides*, MZUEL 7453, 54.6 mm SL. Specimen collected in the Arroio Divisa, Camaquã River basin, Cristal, RS, 30^o^54'5.6"S 52^o^05'18.9"W (Photo by O.A. Shibatta).

**Fig 2:**
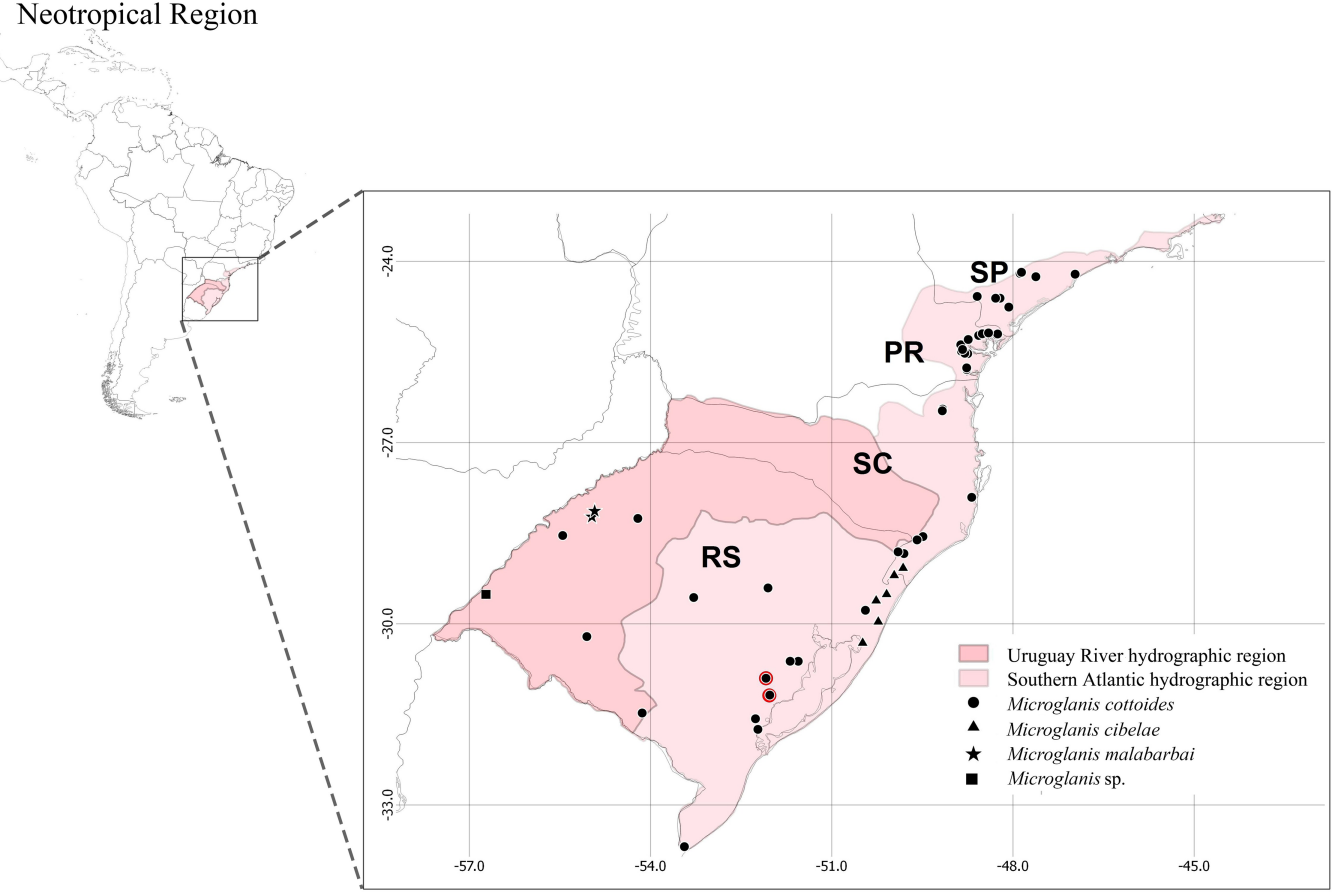
Map with collection points of *Microglanis* in the southeastern and southern states of Brazil. The acronyms SP, PR, SC and RS, refer to the states of São Paulo, Paraná, Santa Catarina and Rio Grande do Sul, respectively. The red circles correspond to the samples from Camaquã river basin, drainage of the type locality of *M*. *cottoides*.

To test the monophyly of *Microglanis cottoides* (Fig 1) we sampled populations across the entire distribution of the species. We collected tissue samples from 81 specimens of *Microglanis cottoides* from eight populations circumscribed to two hydrographic regions (Southern Atlantic and Uruguay River) from São Paulo to Rio Grande do Sul (Fig 2): Ribeira de Iguape–RIB, n = 10; Paranaguá –PAR, n = 11; Guaratuba–GUA, n = 08; Itapocu–ITA, n = 08; Madre–MAD, n = 05; Araranguá –ARA, n = 10; Laguna dos Patos–PAT, n = 20; and Uruguay–URU, n = 9.

In addition, we included samples of *Microglanis* species occurring in sympatry with *Microglanis cottoides* or distributed in the Southern Atlantic and Uruguay basins: *M*. *cibelae* (n = 18), *Microglanis* sp. (n = 4), and *M*. *malabarbai* (n = 6). The species identifications were made based on the external morphology, according to Shibatta & Benine [53], Malabarba & Mahler-Jr. [9], and Bertaco & Cardoso [54]. When only the tissues were available, we followed the identifications of collectors. For *M*. *garavelloi*, COI sequences were obtained from GenBank (JN989032, GU701443, GU701629, GU701627, and GU701626), and included only species from the Upper Paraná River basin. New sequences generated in this study were submitted to GenBank (*M*. *cottoides*: RIB- KP063067 to KP063071, PAR- KP063072 to KP063075, GUA- KP063073 to KP063079, ITA- KP063080 to KP063083, MAD- KP063084 to KP063088, ARA- KP063089 to KP063093, PAT- KP063101 to KP063105, and URU- KP063059 to 063066; *M*. *cibelae*: MAM- KP063094 to KP063095, TRA- KP063096 to KP063100; *M*. *malabarbai*: MF045829 to MF045833; *M*. *parahybae*: KP0603106 to KP063110). Tissue samples of *Microglanis* sp. from Uruguay River basin were obtained by donation and the species identification were maintained as appointed by the collector, because they were from other localities than the type-locality of *M*. *malabarbai*. These specimens were included in the analysis to verify if they could belong to the *M*. *malabarbai* species. Although *M*. *eurystoma* is also distributed in the Uruguay River basin, this species was not included in the molecular analyses as it was not possible to obtain samples from the type locality. *Microglanis parahybae* occurs north of the distribution of *M*. *cottoides* in coastal drainages of the Paraíba do Sul basin. Thus, we included five samples of this species to test the phylogenetic relationship between species sharing a similar environment but occurring in allopatry. Our analyses included a total of 119 sequences. All species used, as well as information on them, are summarized in S1 Table.

DNA extraction from tissues followed a phenol/chloroform protocol [55]. Partial sequences of the mitochondrial gene COI were amplified using polymerase chain reaction (PCR) with the primers FishF1-5’TCA ACC AAC CAC AAA GAC ATT GGC AC-3’ and FishR1-5’AGA CTT CTG GGT GGC CAA AGA ATC A-3’ [56]. We used 15 μl as a total volume containing 7.5 μl GoTaq Green Master Mix (Promega), 0.15 μl of each primer at 20 μM, 2.0 μl genomic DNA (5 ng/μl), and 5.2 μl of double-distilled water. The PCR consisted of an initial denaturation (5', at 95° C) followed by 35 cycles of chain denaturation (30s at 94° C), primer hybridization (54° C), and nucleotide extension (30s at 72° C). PCR products were checked by electrophoresis in agarose gel, purified using EXOSAP (Exonuclease I and Shrimp Alkaline Phosphatase GE Healthcare®, Piscataway, USA) and sequenced in both directions using Big Dye Terminator v 3.1 (Applied Biosystems), with subsequent reading on an automatic sequencer ABI Prism 3500 XL (Applied Biosystems).

#### Alignment, genetic distance, phylogenetic estimation, and species delimitation analyses

Electropherogram Quality Analysis software [57] was used to produce consensus sequences for each individual, from the sequences of both primers. Subsequently, these sequences were edited and aligned using the ClustalW algorithm in MEGA 6.0 [58]. This same software was used to calculate the genetic distance within and between populations using the Kimura-2-Parameters (K2P) model [59]. Traditionally, a threshold of 2% genetic distance has been used to delimit species using COI [49], [38], [41], [60], [61]. Herein we assume this threshold and also estimated the phylogenetic relationships of samples to test the monophyly of nominal species.

The software BEAST v.2.1.3 [62] was used to estimate a Bayesian phylogenetic tree. Fossil data show that the divergence time between *Lophiosilurus alexandri* and *Cephalosilurus apurensis*, both in the Pseudopimelodidae family, is estimated between 15.9 and 11.5 million years (Ma) [63]. We time-calibrated the phylogeny using a node-age approach based on divergence times of these two genera, since *Microglanis* species have not been included previously in time-calibrated phylogenies. We applied a strict clock model with a uniform prior, which is a generally well-justified prior within a species or among a few closely related species [64]. We used as tree prior the Birth-Death model, which is an extension of the Yule model and assumes that at any point in time each lineage may undergo speciation or extinction [65]. The data were analyzed as a single partition and the evolutionary model used was GTR+Γ+I, as specified by the program MrModeltest 2.3 [66]. In BEAST, we ran the analyses for ten million generations, sampling every 1,000th step. The convergence was assessed in Tracer 1.5 with 25% burn-in [67], and we summarized the MCMC samples using the maximum clade credibility topology using TreeAnnotator v1.5.3 [68]. The distribution of haplotypes and mutational step numbers was generated using the software package Network 4.1.0.8 (www.fluxus-engineering.com), with the median-joining method (MJN) [69].

From the results of the phylogenetic analysis, the species delimitation test was performed using the Generalized Mixed Yule Coalescent (GMYC) model [51], which is more suitable for data with one gene. This analysis was conducted using the "Species Limits by Threshold Statistics" package [70], implemented in R Core Team v3.0.1 [71]. By means of this package it is possible to calculate the number of clusters by classifying the bifurcation rates of a phylogeny as a result of interspecific or intra-specific branching processes [51].

### Morphological analyses

A total of 21 linear measurements was obtained using a digital caliper according to Malabarba and Mahler-Jr [9], and Bertaco and Cardoso [54], with addition of the following variables: snout length (measured from the tip of the snout to the base of the anterior eye margin), pelvic fin length, posterior cleithral process length (measured from the origin of the elevated pectoral spine to its osseous tip), dorsal-fin to adipose-fin distance (measured between the posterior base of the dorsal fin and the anterior base of the adipose fin), anus to anterior anal fin base distance, posterior nostrils distance, anterior to posterior nostrils (measured between the posterior base of anterior nostril to anterior base of posterior nostril).

Specimens identified as *Microglanis cottoides*, *M*. *cibelae* and *M*. *malabarbai*, and belonging to the type-locality basins were included in the analysis in order to test the morphological similarity and its congruence with molecular analysis. Variables of body were presented as proportions of standard length (SL) and variables of head were presented as proportions of head length (HL). Differences in average body proportions were tested by One-way ANOVA. Principal components analysis (PCA) on covariance matrix of log transformed data was used to test for morphometrics differences between species, and obtain discriminant characters. Counts of lateral line pores were made on the left side of body, whenever possible. The box plot of counts was built with PAST [72], as well as the ANOVA and PCA were performed with this program.

## Results

### Molecular analyses

#### Barcode and genetic distance

A total of 620 base pairs (bp) of the COI gene were analyzed. We did not find insertions, deletions, or stop codons in these sequences, indicating that all amplified regions correspond to a functional portion of the COI gene.

Genetic distance within the populations considered as *Microglanis cottoides* (RIB, GUA, PAR, ITA, MAD, ARA, PAT, and URU) ranges from 0% to 7%. Genetic distances within coastal drainage populations (RIB, GUA, PAR, ITA, MAD, ARA, PAT) ranges from 0 to 0.4% (Table 1). In contrast, the population of the Uruguay River basin presented the greatest genetic distance relative to the coastal drainages, varying from 5.7% to 7.3%. These distances were even greater than those found between *M*. *cottoides* (coastal drainage) and *M*. *parahybae* (from 3.7% to 4.2%). The latter species occurs north of the distribution of *M*. *cottoides*, in the Paraíba do Sul River basin. Populations of *M*. *cottoides* of the Uruguay River basin occur in sympatry with *M*. *malabarbai* and the genetic distance between them was 0.7%. Low genetic divergences were also observed between *M*. *cibelae* and *M*. *cottoides* (0.5% to 1.8%).

**Table 1:** Mean genetic distances using the K2P model (Kimura-2-parameters) among populations of *Microglanis cottoides* and other species of *Microglanis* obtained with COI data of 119 individuals.

	Estimates of evolutionary divergence over sequence pairs														
	Between Groups/Species													Within Groups	
	1	2	3	4	5	6	7	8	9	10	11	12	13	d	S.E
1. *M*. *cottoides*-RIB		0.003	0.003	0.003	0.004	0.005	0.005	0.012	0.005	0.012	0.011	0.009	0.013	0.000	0.000
2. *M*. *cottoides*-GUA	0.007		0.000	0.000	0.003	0.004	0.004	0.011	0.005	0.011	0.010	0.009	0.013	0.000	0.000
3. *M*. *cottoides*-PAR	0.007	0.001		0.000	0.003	0.004	0.004	0.011	0.005	0.011	0.010	0.009	0.013	0.001	0.001
4. *M*. *cottoides*-ITA	0.007	0.000	0.001		0.003	0.004	0.004	0.011	0.005	0.011	0.010	0.009	0.013	0.000	0.000
5. *M*. *cottoides*-MAD	0.010	0.007	0.007	0.007		0.003	0.003	0.011	0.003	0.011	0.011	0.008	0.012	0.000	0.000
6. *M*. *cottoides*-ARA	0.017	0.013	0.014	0.013	0.008		0.003	0.010	0.004	0.010	0.010	0.008	0.011	0.004	0.002
7. *M*. *cottoides*-PAT	0.015	0.012	0.013	0.012	0.005	0.009		0.011	0.003	0.011	0.010	0.008	0.012	0.002	0.001
8. *M*. *cottoides*-URU	0.073	0.065	0.065	0.065	0.065	0.061	0.066		0.010	0.002	0.003	0.011	0.009	0.003	0.000
9. *M*. *cibelae*	0.018	0.015	0.015	0.015	0.008	0.011	0.005	0.061		0.010	0.010	0.008	0.012	0.001	0.001
10. *Microglanis* sp.	0.071	0.063	0.063	0.063	0.063	0.059	0.064	0.003	0.059		0.004	0.011	0.009	0.000	0.000
11. *M*. *malabarbai*	0.068	0.060	0.060	0.060	0.063	0.058	0.064	0.007	0.059	0.010		0.011	0.009	0.002	0.001
12. *M*. *parahybae*	0.041	0.042	0.042	0.042	0.038	0.040	0.037	0.069	0.041	0.067	0.067		0.012	0.000	0.000
13. *M*. *garavelloi*	0.079	0.075	0.076	0.075	0.071	0.067	0.069	0.045	0.071	0.043	0.050	0.071		0.000	0.000

Standard error estimates are shown above the diagonal between groups/species.

#### Haplotype network

**Fig 3:**
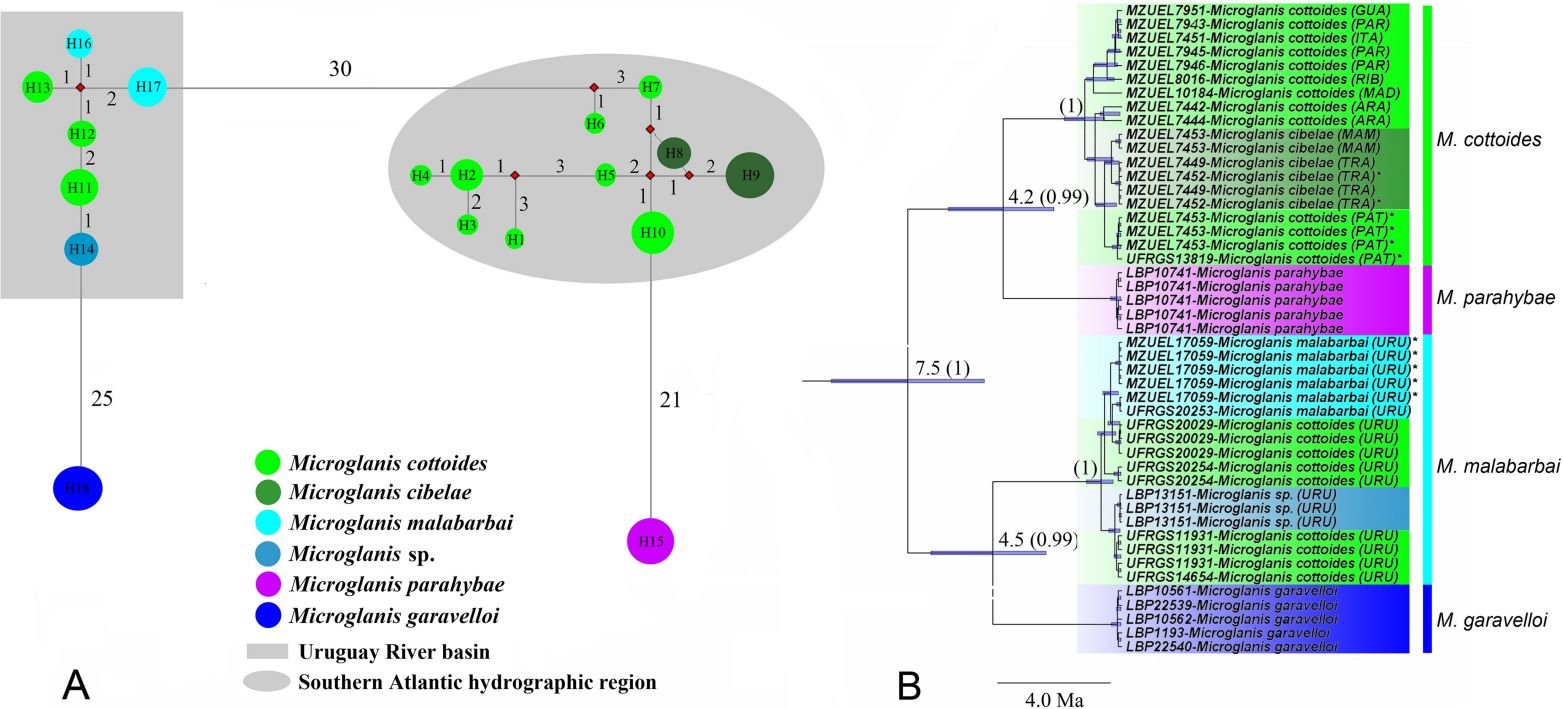
Median-joining networks and Bayesian phylogenetic tree of *Micriglanis* obtained with COI data. (A) Median-Joining networks among haplotypes. Each circle represents a unique haplotype with circle sizes being proportional to their frequencies. Each color represents a species. The numbers between haplotypes correspond to mutational steps. Haplotypes: H1 = Ribeira; H2 = Guaratuba + Paranaguá + Itapocu; H3 = Paranaguá; H4 = Paranaguá; H5 = Madre; H6 = Araranguá; H7 = Araranguá; H8 = *M*. *cibelae* (Mampituba); H9 = *M*. *cibelae* (Tramandaí); H10 = Patos (Camaquã); H11 = Negro + Uruguay; H12 = Uruguay; H13 = Uruguay; H14 = *Microglanis* sp. Uruguay; H15 = *M*. *parahybae*: H16 = *M*. *malabarbai*; H17 = *M*. *malabarbai*; H18 = *M*. *garavelloi*. (B) Bayesian tree. Node bars represent the threshold time for each cladogenetic event. The values above the branches indicate the posterior probability (pp). * Individuals collected in their respective type locality.

We found 18 haplotypes among samples of *Microglanis* in Southern Brazil (Fig 3A). *Microglanis cottoides* from the Uruguay River basin grouped with *Microglanis* sp., and *M*. *malabarbai*, whereas *M*. *cottoides* from the coastal drainages grouped with *M*. *cibelae*. The network shows the presence of two distinct groups within *M*. *cottoides*, separated by 30 mutational steps (i.e. Uruguay River basin and coastal basins). The number of mutational steps separating individuals from these two regions was greater than that found between *M*. *parahybae* and *M*. *cottoides* (21 mutational steps). However, the number of mutational steps found between *M*. *malabarbai* and populations of *M*. *cottoides* from the Uruguay River basin (one to two mutational steps) was similar to the number of mutations between *M*. *cottoides* populations of the coastal basins (Fig 3A).

#### Phylogenetic analyses and GMYC

The topology of the Bayesian phylogeny (Fig 3B, completed tree shown as S1 Fig) does not support the monophyly of *Microglanis cottoides*. Although most of the samples are grouped in the same clade with a high posterior probability (pp = 1), *M*. *cibelae* is nested within samples of *M*. *cottoides*. Furthermore, populations of *M*. *cottoides* from the Uruguay River basin are paraphyletic, since they are clustered with *M*. *malabarbai*.

In the molecular phylogeny, we recovered *Microglanis parahybae* as sister to *M*. *cottoide*s, whereas *M*. *garavelloi* is phylogenetically closer to *M*. *malabarbai*. The coastal species *M*. *parahybae* and *M*. *cottoides* diverged about 4.2 Ma and the species in the Uruguay River basin *M*. *garavelloi* and *M*. *malabarbai* diverged about 4.5 Ma (Fig 3B). These two major clades of coastal and inland species diverged about 7.5 Ma (Fig 3B). Two large clades are observed in *M*. *cottoides*, one in the southern drainages from the Araranguá River to Laguna dos Patos drainage and the other in northern drainages including Ribeira de Iguape, Paranaguá, Guaratuba, Itapocu, and Madre river drainages (Fig 3B).

The GMYC analysis suggests the presence of four evolutionary independent lineages within the samples included here. *Microglanis cottoides* comprise two clusters: Cluster 1, samples of the coastal basins + *Microglanis cibelae*, and cluster 2, samples of Uruguay river basin + *M*. *malabarbai*. Moreover, *M*. *parahybae* and *M*. *garavelloi* were identified as separated lineages in the analysis (S2 Fig).

### Morphological analyses

The samples examined showed similar SL mean (Tab. 3; ANOVA: F = 0.3736, p = 0.6904). Regarding body proportions, the minimum and maximum values of all variables overlapped, preventing the prompt identification of species (Table 2). Only head depth mean differed from all species (ANOVA: F = 52.59, p < 0.001). Regarding *M*. *cottoides* and *M*. *cibelae*, we identified differences in snout length (ANOVA: F = 7.156, p = 0.002). *Microglanis malabarbai* show great morphological disparity from the others species, particularly related to: orbital diameter (F = 16.17, p < 0.001), dorsal-fin spine length (F = 11.97, p < 0.001), pectoral-fin spine length (F = 31.4, p < 0.001), predorsal length (F = 11.23, p < 0.001), dorsal-fin base length (F = 4.858, p = 0.01241), anus to anal fin distance (F = 9.392, p < 0.001), anterior nostrils distance (F = 10.65, p < 0.001). The variables that, on average, differentiate only *M*. *malabarbai* and *M*. *cibelae* were: head length (F = 4.402, p = 0.01809), mouth width (F = 3.055, p = 0.05722), maxillary barbel length (F = 7.517, p = 0.001555), adipose-fin base length (F = 8.885, p < 0.000574), and anal-fin base length (F = 3.473, p = 0.03978). The variables that differ only *M*. *malabarbai* and *M*. *cottoides* were: body depth (F = 5.368, p = 0.008202), dorsal to adipose fin distance (F = 3.556, p = 0.03701), anterior to posterior nostrils distance (F = 3.957, p = 0.02628), and caudal peduncle length (F = 3.852, p = 0.2875). The variables that did not differentiate between the species were: interobital width (F = 0.9327, p = 0.4011), pelvic fin length (F = 0.367, p = 0.6949), posterior cleithral process length (F = 0.2766, p = 0.7597), prepelvic length (F = 2.666, p = 0.08074), preanal length (F = 1.013, p = 0.3716), caudal peduncle depth (F = 2.116, p = 0.1326), posterior nostrils distance (F = 0.3461, p = 0.7094).

**Table 2:** Morfometry of *Microglanis cottoides* (n = 19), *M*. *cibelae* (n = 15) and *M*. *malabarbai* (n = 13).

	*M*. *cottoides* (n = 14)		*M*. *cibelae* (n = 15)		*M*. *malabarbai* (n = 13)	
	Min-max	Mean±SD	Min-max	Mean±SD	Min-max	Mean±SD
Standard length (mm)	28.8–63.6	42.2±9.5	30.5–60.2	42.8±8.4	23.8–52.7	39.8±11.4
**Proportions of standard length**						
Head length	26.7–32.7	28.6±1.3	26.4–29.3	28.0±1.0	26.6–33.9	29.6±1.9
Pelvic fin length	15.7–20.0	18.2±1.2	15.6–19.9	18.3±1.4	15.8–22.4	18.7±2.0
Dorsal-fin spine length	11.9–18.2	15.3±1.7	13.8–19.6	16.7±1.7	11.8–15.9	13.8±1.2
Pectoral-fin spine length	16.4–23.6	21.0±1.9	19.7–25.5	22.5±1.6	12.5–20.7	16.9±2.3
Posterior cleithral process length	14.3–18.4	16.0±1.2	13.0–17.8	16.0±1.2	14.2–17.3	15.8±1.0
Predorsal length	35.1–38.6	36.9±0.9	34.5–39.1	36.1±1.3	36.7–39.3	38.0±0.9
Prepelvic length	48.5–55.6	52.1±1.9	48.2–53.3	50.8±1.6	49.1–52.9	51.0±1.3
Preanal length	65.3–72.4	69.5±1.8	50.5–71.6	68.0±5.1	67.2–71.2	69.1±1.3
Caudal peduncle depth	8.9–11.9	10.4±0.7	8.6–12.4	10.6±1.0	8.0–10.9	9.9±0.8
Caudal peduncle length	14.0–19.5	16.6±1.3	13.7–17.2	15.7±1.1	13.4–18.1	15.5±1.3
Body width	27.0–32.7	30.1±1.7	25.7–30.8	28.1±1.3	28.4–31.8	30.0±1.1
Body depth at anal-fin origin	16.0–20.9	17.6±1.4	14.1–19.7	17.2±1.7	13.8–18.5	15.9±1.4
Dorsal-fin base length	12.3–16.8	14.6±1.1	13.8–16.6	14.8±0.7	11.6–15.1	13.7±0.9
Adipose-fin base length	17.8–25.9	20.5±2.1	19.5–25.3	22.2±1.8	16.7–23.1	19.1±1.9
Anal-fin base length	13.3–17.9	14.9±1.2	13.9–18.0	15.5±1.2	12.6–16.4	14.3±1.3
Dorsal-fin to adipose-fin distance	13.0–20.5	17.4±2.0	15.3–21.2	17.5±1.6	16.3–21.7	19.0±1.7
Anus to anterior anal-fin base distance	6.7–10.4	8.1±1.2	6.4–11.3	8.2±1.3	8.6–11.9	9.8±1.1
**Proportions of head length**						
Head width	59.7–84.3	70.8±6.4	70.9–93.3	80.3±5.6	52.6–67.0	64.2±3.9
Head depth	53.5–75.9	66.1±5.6	69.3–97.9	83.1±9.3	48.1–62.6	58.2±3.6
Interorbital width	39.3–49.5	44.4±2.4	40.6–48.6	44.5±2.1	34.8–51.5	45.7±4.2
Orbital diameter	8.0–12.1	10.1±1.0	9.0–13.0	10.7±1.0	7.4–9.8	8.7±0.9
Snout length	31.1–40.9	37.4±2.5	38.7–43.0	40.6±1.3	30.8–41.8	38.5±3.4
Mouth width	44.4–64.1	54.3±5.4	48.1–58.7	52.9±3.6	40.4–65.5	57.8±6.8
Maxillary barbel length	76.3–117.5	97.2±11.2	77.3–123.7	103.0±10.8	63.0–105.2	85.8±13.7
Anterior nostrils distance	23.0–28.5	26.1±1.5	25.1–30.1	26.7±1.3	22.4–32.7	29.2±3.0
Posterior nostrils distance	30.2–37.4	34.7±1.9	26.9–37.1	34.0±2.7	26.3–38.0	34.4±2.8
Anterior to posterior nostrils distance	17.2–25.6	20.3±2.1	17.0–22.1	20.0±1.4	13.8–23.4	18.4±2.3

**Fig 4:**
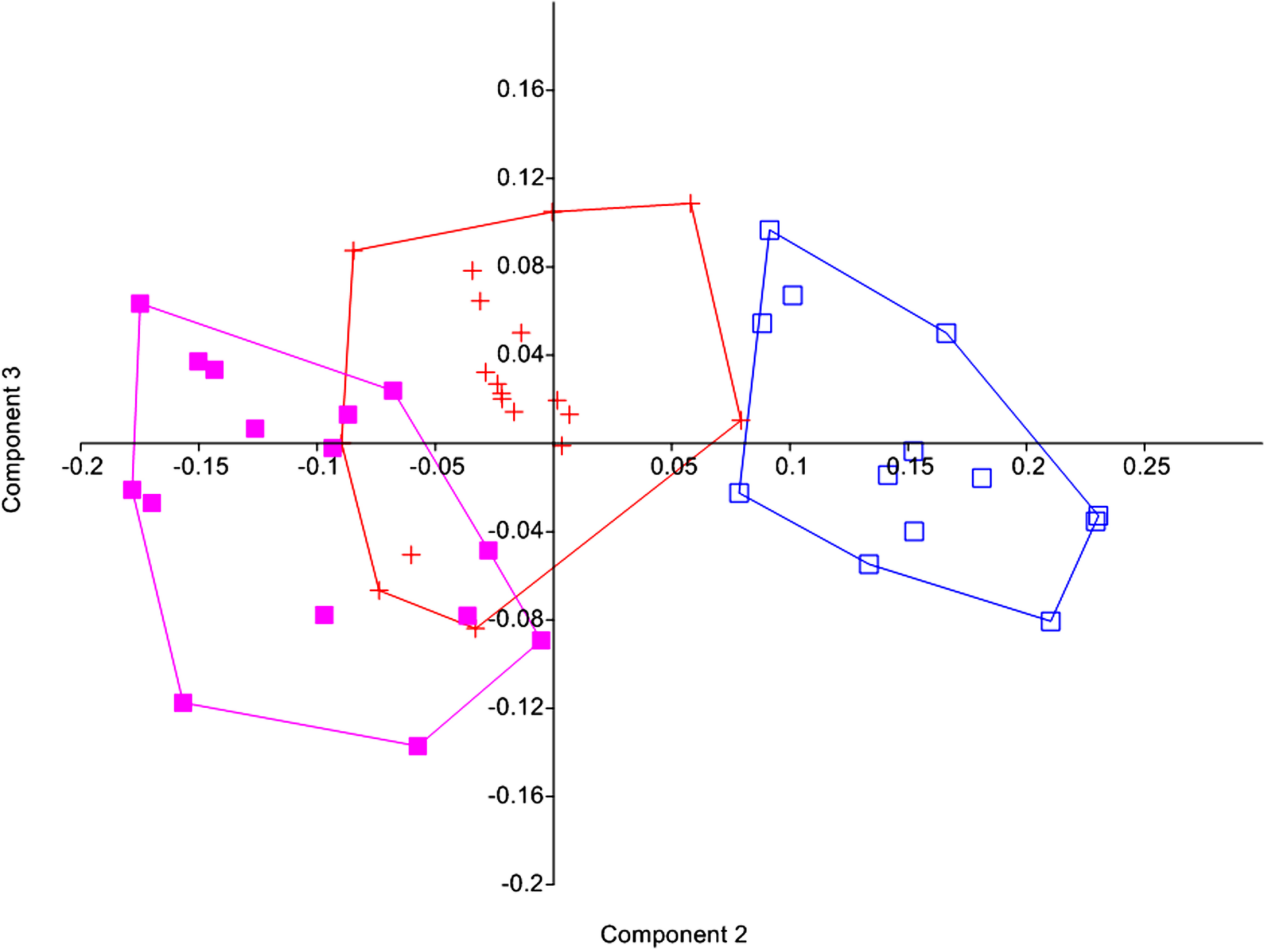
Dispersion of individual scores on the second and third Principal Components axis. *Microglanis cottoides* (red, n = 19), *M*. *cibelae* (pink, n = 15) and *M*. *malabarbai* (blue, n = 13).

The Principal components analysis of the combined samples of *Microglanis cottoides*, *M*. *cibelae* and *M*. *malabarbai* showed better separation of the groups with components 2 and 3 (Fig 4). The first component, which retained most of the variation (89.4%), was the representative of size. The second and third components retained 3.8% and 1.0%, respectively, and best represented the shape. In PC2, *Microglanis malabarbai* differs from *M*. *cibelae*, for presenting longer maxillary barbel length, anterior internarial distance, prepelvic length, and mouth width (positive variable loadings, Table 3), and for smaller body width, dorsal-fin spine length, preanal length, posterior cleithral process length, and caudal peduncle length (negative variables loadings, Table 3). In this axis, *M*. *cottoides* is in intermediary position, not separating from *M*. *cibelae*, and evidencing greater similarity with *M*. *cibelae* than with *M*. *malabarbai*.

**Table 3:** Variables loadings on first three principal components axis of cobined samples of *Microglanis cottoides*, *M*. *cibelae*, and *M*. *malabarbai*.

	Axis 1	Axis 2	Axis 3
Standard length	0.1806	0.04863	0.03859
Head length	0.1736	0.1387	-0.07296
Pelvic fin length	0.2019	-0.1664	-0.1344
Dorsal-fin spine length	0.2219	**-0.3401**	-0.1911
Pectoral-fin spine length	0.1894	0.1785	-0.1273
Posterior cleithral process length	0.1821	**-0.2169**	-0.1345
Predorsal length	0.1921	0.08389	-0.2405
Prepelvic length	0.2076	**0.2876**	0.05332
Preanal length	0.1644	**-0.224**	0.3391
Caudal peduncle depth	0.1542	0.02892	0.2447
Caudal peduncle length	0.1973	**-0.211**	-0.4491
Body width	0.2396	**-0.3713**	-0.1262
Body depth anal origin	0.2011	0.06361	-0.08816
Dorsal-fin base length	0.1748	0.1207	-0.03506
Adipose-fin base length	0.195	0.06557	0.01681
Anal-fin base length	0.1908	0.1001	0.09773
Dorsal to adipose	0.1927	-0.06196	0.05217
Anus to anterior anal-fin base distance	0.1741	-0.02244	0.3133
Head width	0.1978	0.1418	0.1002
Head depth(altura)	0.2217	-0.05145	0.2617
Interorbital width (largura)	0.1887	-0.0532	0.04163
Orbital diameter	0.2015	-0.1947	0.09129
Snout length (comprimento)	0.1707	-0.109	0.2734
Mouth width	0.1858	**0.2405**	0.2472
Maxillary barbel length	0.1162	**0.3776**	-0.2357
Anterior nostrils distance	0.1843	**0.3017**	-0.2231
Posterior nostrils distance	0.1884	0.167	0.001884
Anterior to posterior nostrils distance	0.1647	-0.03168	-0.04127
Eigenvalue	0.319649	0.0125636	0.00343709
% variance	89.3	3.5	1.0

Variables with higher loadings are in bold.

#### Lateral line pore counts

**Fig 5:**
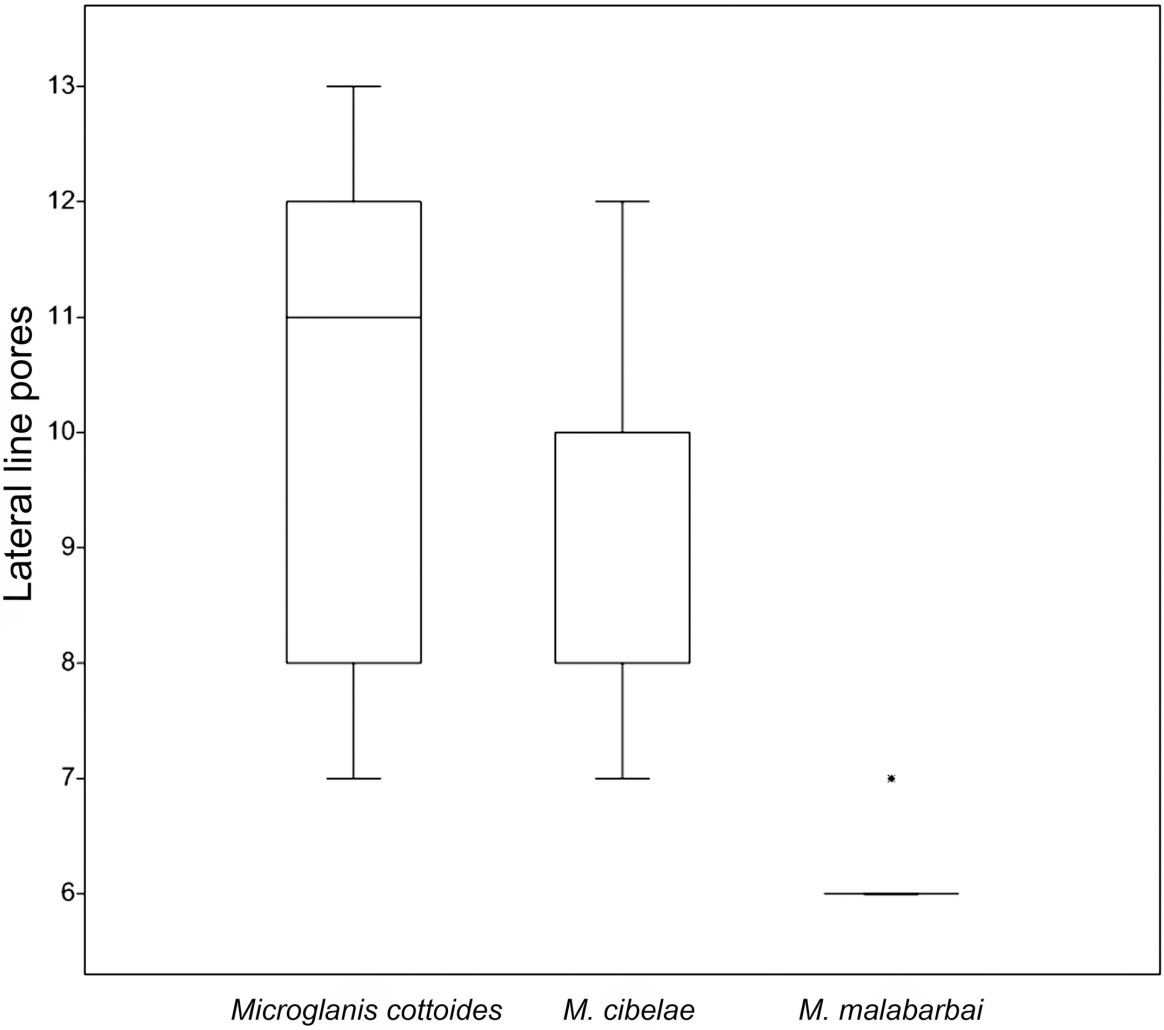
Box plot of the lateral line pore counts. *Microglanis cottoides* (n = 17), *M*. *cibelae* (15), and *M*. *malabarbai* (n = 12). Point represents outlier.

Lateral line pore counts showed overlap in *Microglanis cottoides* (7–13, median = 11, n = 17) and *M*. *cibelae* (7–12, median = 10, n = 15), but differed *M*. *malabarbai* (6–7, median = 6, n = 12) (Fig 5).

## Discussion

### Molecular approach

*Microglanis cottoides* was originally described from the Camaquã River, a tributary of the Laguna dos Patos basin, but its distribution was extended to the Uruguay River basin [9], and the coastal rivers of São Paulo to Rio Grande do Sul States [23]. However, the molecular evidence of our study strongly suggests that *M*. *cottoides*, as currently defined, does not form a monophyletic group, since the population occurring in the Uruguay River basin is genetically distinct, and *M*. *cibelae* is nested within *M*. *cottoides*.

The genetic distance between populations of *Microglanis cottoides* from the coastal basins and the Uruguay River basin (5.6% to 7.0%) corroborate the hypothesis that the Uruguay River population of *M*. *cottoides* has been misidentified. For instance, divergences of this magnitude are often found among congeners of other Neotropical fish [37], [39], [41]. In addition, our results also demonstrate no overlap between the intra and interspecific distances (barcoding gap) of the individuals from the Uruguay River basin and the coastal basins, as well as with the other species of *Microglanis* added to the analysis (except for *M*. *cibelae*) confirming the safe discrimination of species using the barcoding methodology suggested by Hebert *et al*. [30] and Meyer & Pauly [73]. Thus, although others species of fish occur in both the coastal basins and the Uruguay basins [74], [75], [76], [77], it is not the case of *M*. *cottoides*, which has its distribution restricted to coastal drainages.

On the other hand, the low genetic distance between populations preliminarily identified as *M*icroglanis *cottoides* and *Microglanis* sp. from the Uruguay River basin and *M*. *malabarbai* (0.1% to 0.6%), and between *M*. *cottoides* and *M*. *cibelae* (0.5% to 1.8%) from the coastal drainages, agree with intra-population variances observed within other species [39], [41], [45] [78], [79].

The haplotype network corroborates the results observed both in phylogenetic analysis and genetic distances. According to Hudson *et al*. [80], the haplotypes represent the nodes in a group of closely related taxa, linked to each other according to the similarity of the haplotype sequences. Sequence divergence in *Microglanis* were lower within species than between closely related species, except for *M*. *cibelae*. Also, in the network is possible to visualize two very distinct haplogroups within *M*. *cottoides* (Uruguay River, corresponding to *M*. *malabarbai*, *vs*. coastal basins), separated by a high number of mutational steps, equivalent to those found among the other species of *Microglanis* added in the analysis. In fact, it is larger than that found between the haplogroups of *M*. *cottoides* from the coastal basin and *M*. *parahybae* from the Paraíba do Sul River basin.

The GMYC allows the calculation of the number of clusters resulting from interspecific or intraspecific branching processes [51] and this analysis indicated two clusters for *Microglanis cottoides*, containing the same clades recovered in the phylogenetic analysis, which reinforces that the species known as *M*. *cibelae* is a junior synonym of *M*. *cottoides* and that individuals from the Uruguay River basin, identified as *M*. *cottoides*, are actually *M*. *malabarbai*. In addition, this tool has a tendency to overestimate the number of lineages [51] in other words, to identify intraspecific lineages as distinct lineages, which did not occur with *M*. *cibelae*, reinforcing the hypothesis of synonymy.

The phylogenetic analysis showed that the coastal species *Microglanis cottoides* and *M*. *parahybae* are closely related and have diverged about 4.2 Ma; *Microglanis garavelloi* from the Upper Paraná River basin and *M*. *malabarbai* from the Uruguay River diverged also in the Pliocene and have been separated from the coastal species since the late Miocene (± 7.5 Ma). These results agree with Weitzman & Weitzman [81] and Lundberg [82], who suggest that diversification of fish up to the level of modern species predates the Pleistocene, as Albert & Reis [83] state that most Neotropical ichthyofauna diverged between 3.0 and 10 Ma.

The origin of many southern and southeastern Brazilian drainages that now run directly into the Atlantic Ocean was at the time of tectonic activity at the beginning of Tertiary (65–1.8 Ma), generating a complex system of 'failures' in the crystalline shield that culminated in erosions and subsequent events of headwater captures, as occurred between the Tietê River, of the Upper Paraná River basin, and the Paraíba do Sul River [1]. These events were also responsible for the distribution of some species into neighboring drainages, such as Ribeira de Iguape, as well as smaller coastal drainages [84], [85], [86], [87], [88]

The differentiation of species by geographic isolation is the reflection of the geological past and the environmental changes occurring in the region [1], [89], and the degree of genetic divergence is strongly correlated with the age of physical isolation [90]. Thus, the tectonic activity that began in the Tertiary, pobably allowed the species of *Microglanis* of Southeast-South coast first diverge of the species of the Upper Paraná River system (± 7.5 Ma). Later, in a more recent episode (± 4.2 Ma) that likely involved the Serra do Mar, the diversification of coastal species occurred, separating *M*. *cottoides* (from São Paulo to Rio Grande do Sul States), from *M*. *Parahybae* (from Rio de Janeiro).

Species of fishes with similar distribution of *Microglanis cottoides* have already been observed south of Rio de Janeiro. For example, *Cyphocharax santacatarinae* (Fernández-Yépez 1948) occurs from Santa Catarina to São Paulo, in the Ribeira de Iguape River [91] and *Oligosarcus hepsetus* (Cuvier 1829) occurs in coastal rivers from Santa Catarina to Rio de Janeiro [92]. The comparison of inter and intraspecific patterns from co-distributed species allows the evaluation of how independent lineages have responded to the same historical processes in a given region [93], [94]. In this way, it is possible to suggest that this region served as an important ichthyofauna divisor of the rivers located to the north and to the south.

At the time of the separation between the coastal species and those of the adjacent plateau, the Uruguay River maintained a connection with what is now called the Upper Parana River and, according to Beurlen (*apud* [95]), only came to separate in the Miocene (24–5.3 Ma) on occasion of erosion in its basaltic cover. The close phylogenetic relationship between *Microglanis garavelloi* of the Upper Paraná River and *M*. *malabarbai* of the Uruguay River corroborates this fact.

The shared distribution of species among isolated basins may reflect recent vicariant events, such as changes in the drainage course or capture of rivers from one basin by the other, generated by geomorphological modifications [1]. Alternatively, in the case of *Microglanis cottoides*, dispersion between coastal rivers, due to the sea level fluctuations in the late Pleistocene, mainly by marine regression in the glacial periods, that allowed communication between drainages along the continental shelf [96] [97]. In the Brazilian continental shelf, recent studies have shown that sea level fluctuations during the Quaternary have left evidence, such as paleodrainages, that confirm successive stages of exposure and submersion [2], [98], [99], [100], [101]. Thus, geomorphological features associated with fluvial channels on continental shelves may indicate that coastal drainage, which now flows directly into the oceans, had communication with other nearby drainage basins [101], allowing ichthyofauna flow. This is one of the plausible explanations for the wide distribution of many species, including *M*. *cottoides*, in coastal drainages that are now isolated.

### Morphological approach

Malabarba & Mahler-Jr. [9] diagnosed *Microglanis cibelae* for having a smaller head than *M*. *cottoides* (25.6–31.1% SL vs. 29.3–33.8%), and lower body width (25.4–29.8% SL vs. 28.5–33.9%). As can be observed there were overlaps in the proportions, which does not allow the species to be safely identified. In our analyses we also did not find intervals of these characteristics that could differentiate the samples as distinct species and, therefore, we used the PCA, because it was considered more suitable to measure the variation of the morphometric variables [102]. Nevertheless, there was a wide overlap between *M*. *cottoides* and *M*. *cibelae* on the second principal component axis. Malabarba & Mahler-Jr. ([9], p.253) still presented a linear regression graph pointing to ontogenetic divergence in head size, which is relatively higher in *M*. *cottoides* than in *M*. *cibelae* in specimens larger than 30 mm SL. The same was observed in our study, corroborating those authors [9]. However, these variations in morphological characteristics may represent different populations, not different species. Malabarba & Mahler-Jr. [9] also include samples from the Uruguay River basin identified as *M*. *cottoides*. By our analysis, all the samples identified as *M*. *cottoides* of this basin correspond to the species *M*. *malabarbai*, whose differences are very large in relation to *M*. *cibelae*, and which may have influenced the decision of those authors.

Initially, the possibility of a cryptic species in sympatry with *Microglanis malabarbai* was considered, but was not confirmed, as the genetic divergence is too low to separate them into distinct species. However, a more complex situation seems to be involved, considering the two reviews of *Microglanis* from southern Brazil [9], [54]. Both studies, analyzing only the morphology, were not able to distinguish some populations of *Microglanis* from the Uruguay and Laguna dos Patos basins, even with high genetic divergence among them observed herein, raising the hypothesis that the morphological variation in populations of *M*. *cottoides* and *M*. *malabarbai* make them difficult to identify correctly. In addition, both papers describe differences in head length, body width, pectoral spine length, maxillary barbel length, and internareal distance to diagnose *M*. *cibelae*, *M*. *cottoides* (including Uruguay River and Laguna dos Patos samples), and *M*. *malabarbai*, indicating the presence of morphological variation among their samples. Both *M*. *cottoides* and *M*. *malabarbai* present strongly structured populations (e.g. *M*. *cibelae* corresponds to a clade among remaining lineages of *M*. *cottoides*) in such a way that morphometric differences described previously may correspond to morphological differences among lineages or populations within each species.

The greater morphological proximity between *M*. *cottoides* and *M*. *cibelae* than with *M*. *malabarbai* observed with PCA, corroborates with the molecular results. Although our analyses point out differences between the head depth proportions of *M*. *cottoides* and *M*. *cibelae*, this measure must be carefully analyzed, as it is altered according to the opening of the mouth. On the other hand, snout length proportions differ significantly between the two samples. However, the wide overlap of the morphometry of *M*. *cottoides* with *M*. *cibelae* on PC2 and PC3 shows that the differences observed in morphological proportions occur at population level. On the other hand, multivariate morphometry corroborates the validity of *M*. *malabarbai*. Among the characters pointed out by Malabarba & Mahler-Jr [9] to distinguish *M*. *cottoides* from *M*. *cibelae*, head length and body width, no significant differences were found in this investigation.

Regarding the lateral line pores, Bertaco & Cardoso [54] also noticed a larger number in *Microglanis cottoides* than in *M*. *malabarbai*. In the description of *M*. *cibelae*, Malabarba & Mahler-Jr. [9] point 7 to 10 pores (usually 8) for the species, a number similar to that of *M*. *cottoides* in this study. With respect to the *M*. *cibelae* specimens examined, a greater variation is observed in this study than that by Malabarba & Mahler-Jr [9], mainly in maximum and modal numbers, but with a similar minimum number. The color pattern is another character pointed by Bertaco & Cardoso [54] differing *M*. *malabarbai* with *M*. *cottoides* and *M*. *cibelae*, but the analysis of samples from different localities of Uruguay River basin has shown variations that generate confusions (Oscar Shibatta, pers. obs.).

Based on our results, we propose that *Microglanis cibelae* is a junior synonym of *M*. *cottoides* and that individuals from the Uruguay River basin, previously identified as *M*. *cottoides*, are actually *M*. *malabarbai*, refuting the initial hypothesis of this work that there are cryptic species of *Microglanis* in the studied coastal drainages, and corroborating that only *M*. *cottoides* occurs in the coastal drainages from São Paulo to Rio Grande do Sul states and does not occur in the Uruguay River basin. Regarding the phylogenetic hypotheses proposed in this contribution, we are planning to test them with new analyses that include more genes.

## Supporting information

S1 Table Taxon, vouchers and locality information of the analyzed specimens of *Microglanis*. *Material examined in the morphological analyses.
(DOCX)

S1 Fig Detailed Bayesian phylogenetic tree of *Microglanis* based on COI data. (TIF)

S2 Fig Bayesian tree obtained through analysis of the COI gene. Branches highlighted in red are the result of interspecific and intra-specific branching processes of *Microglanis* lineages, using the GMYC model, based on the results of the phylogenetic analysis. In particular, *M*. *cibelae* associated to *M*. *cottoides* from coastal drainage and *M*. *cottoides* from the Uruguay River basin, associated with *M*. *malabarbai*. The values above the branches refer to the posterior probability.
(TIF)
