# Supplementary material for: Reappraisal of the systematics of Microglanis cottoides (Siluriformes, Pseudopimelodidae), a catfish from southern Brazil
Source: PLoS One. 2018 Jul 5;13(7):e0199963. doi: 10.1371/journal.pone.0199963 (PMC6033443; doi:10.1371/journal.pone.0199963)
Supplement: S1 Table — *Material examined in the morphological analyses. (DOCX) [file pone.0199963.s001.docx]

S1 Table. Taxon, vouchers and locality information of the analyzed specimens of *Microglanis.**Material examined in the morphological analyses.

| **Taxon** | **Voucher** | **Locality (Drainage/Basin)** | **City, State** | **Geographic coordinates** |
| --- | --- | --- | --- | --- |
| *M. cottoides* | MZUEL 8015 | Rio Ipiranga, Ribeira de Iguape basin (RIB) | Sete Barras-SP | 24º10'49.1"S 47º51'31.8"W |
| *M. cottoides* | MZUEL 8014 | Rio Juquiá, Ribeira de Iguape basin (RIB) | Juquié-SP | 24º11'14"S 47º37'15"W |
| *M. cottoides* | MZUEL 8017 | Rio Preto I, Ribeira de Iguape basin (RIB) | Sete Barra-SP | 24º11'29.8"S 47º53'21.1"W |
| *M. cottoides* | MZUEL 8018 | Rio Preto II, Ribeira de Iguape basin (RIB) | Sete Barra-SP | 24º12'2078"S 47º53'52.3"W |
| *M. cottoides* | MZUEL 8016 | Rio Fau, Ribeira de Iguape basin (RIB) | Miracatu-SP | 24^0^12´44.1”S 47^0^28´61.6”W |
| *M. cottoides* | MZUEL 8021 | Rio Iporanga, Ribeira de Iguape basin (RIB) | Iporanga-SP | 24º34'49"S 48º35'30.2"W |
| *M. cottoides* | MZUEL 8019 | Rio Jaguary, Ribeira de Iguape basin (RIB) | Itapeúna-SP | 24º36'33.8"S 48º12'48.4"W |
| *M. cottoides* | MZUEL 8020 | Rio Batatal, Ribeira de Iguape basin (RIB) | Eldorado-SP | 24º36'34.7"S 48º17'14.2"W |
| *M. cottoides* | MZUEL 7947 | Rio Bananal, Paranaguá basin (PAR) | Paranaguá-PR | 25º11'041"S 48º24'16.5"W |
| *M. cottoides* | MZUEL 7941 | Rio Tagaçaba, Paranaguá basin (PAR) | Guaraqueçaba-PR | 25º11'54.3"S 48º30'25.5"W |
| *M. cottoides* | MZUEL 7942 | Rio Utinga, Paranaguá basin (PAR) | Guaraqueçaba-PR | 25º12'6.1"S 48º15'13.1"W |
| *M. cottoides* | MZUEL 7949 | Rio Ipiranga, Paranaguá basin (PAR) | Paranaguá-PR | 25º12'58.7"S 48º15'58.7"W |
| *M. cottoides* | MZUEL 7950 | Rio Capivari, Paranaguá basin (PAR) | Paranaguá-PR | 25º13'35.2"S 48º34'3.9"W |
| *M. cottoides* | MZUEL 7945 | Rio Mergulhão, Paranaguá basin (PAR) | Antonina-PR | 25º17'35.1"S 48º42'22.1"W |
| *M. cottoides* | MZUEL 7953 | Rio das Antas, Paranaguá basin (PAR) | Guaraqueçaba-PR | 25º17'36.6"S 48º44'25.1"W |
| *M. cottoides* | MZUEL 7943 | Rio Passa Sete, Paranaguá basin (PAR) | Paranaguá-PR | 25º31'32.2"S 48º48'22.8"W |
| *M. cottoides* | MZUEL 7946 | Rio Sambaqui¸ Paranaguá basin (PAR) | Morretes-PR | 25º31'38"S 48º44'56.8"W |
| *M. cottoides* | LBP 2081 | Rio Nhundiaquara, Paranaguá basin (PAR) | Morretes-PR | 25^0^27´29.4”S 48^0^50´05.5”W |
| *M. cottoides* | LBP 762 | Rio Passa Sete, Paranaguá basin (PAR) | Morretes-PR | 25º31´23.1”S 48047´900”W |
| *M. cottoides* | MZUEL 7951 | Rio Canavieira, Guaratuba basin (GUA) | Guaratuba-PR | 25º42'23.3"S48º45'13.2"W |
| *M. cottoides* | MZUEL 7952 | Guaratuba basin (GUA) | Guaratuba-PR | 25º45'46.7"S48º45'58.7"W |
| *M. cottoides* | MZUEL 7944 | Rio Rasgado, Guaratuba basin (GUA) | Guaratuba-PR | 25º47'25.9"S 48º45'57.7"W |
| *M. cottoides* | MZUEL 7441 | Rio Itapocu, Itapocu basin (ITA) | Jaraguá do Sul-SC | 26º26'47.4"S 49º09'49.3"W |
| *M. cottoides* | MZUEL 7451 | Ribeirão Cavalo, Itapocu basin (ITA) | Jaraguá do Sul-SC | 26º28'16.8"S 49º10'56"W |
| *M. cottoides* | MZUEL 10184 | Rio Madre, Madre basin (MAD) | Paulo Lopes-SC | 27^0^54´22”S 48^0^40´57.3”W |
| *M. cottoides* | MZUEL 7442 | Rio Manin, Araranguá basin (ARA) | Siderópolis-SC | 28^0^ 33'22.15"S 49º29'22.5"W |
| *M. cottoides* | MZUEL 7444 | Rio Serrinha, Araranguá basin (ARA) | Siderópolis-SC | 28^0^36'39.5"S 49º35'16.6"W |
| *M. cottoides* | MZUEL 7447 | Rio Serra Velha, Araranguá basin (ARA) | Timbé do Sul-SC | 28^0^48'30.64"S 49º54'6.7"W |
| *M. cottoides* | MZUEL 7446 | Rio Amola Faca, Araranguá basin (ARA) | Timbé do Sul-SC | 28^0^50'15.9"S 49º48'16.2"W |
| *M. cottoides* | MZUEL 6033 | Rio Forquetinha, Laguna dos Patos basin (PAT) | Canudos do Vale-RS | 29^0^24'22.4"S 52º03'19.2"W |
| *M. cottoides* | LBP 14547 | Laguna dos Patos basin (PAT) | Agudos-RS | 29^0^33´53.4”S 53^0^17´08.1”W |
| *M. cottoides* | MZUEL 7450 | Arroio Teixeira, Laguna dos Patos basin (PAT) | Sentinela do Sul-RS | 30^0^37'13.6"S 51º33'17"W |
| *M. cottoides* | UFRGS 13819 | Rio Camaquã, Laguna dos Patos basin (PAT) | Camaquã-RS | 30^0^45´11”S 51^0^38´7.6”W |
| *M. cottoides* | UFRGS 12499 | Arroio Santa Isabel, Laguna dos Patos basin (PAT) | Cristal-RS | 31^0^10´48.3”S 52^0^01´33.8”W |
| *M. cottoides* | MZUEL 7448 | Arroio Contagem, Laguna dos Patos basin (PAT) | Pelotas-RS | 31^0^34'20.15"S 52º12'46.9"W |
| *M. cottoides* | LBP 14517 | Laguna dos Patos basin (PAT) | Chuí-RS | 33^0^41´22.6”S 53^0^26´22.3”W |
| *M. cottoides* | UFRGS 13605 | Arroio Carmelita, Laguna dos Patos basin (PAT) | Pelotas-RS | 31^0^44´52”S 52^0^13´22”W |
| **M. cottoides* | MZUEL 7453 | Rio Camaquã, Laguna dos Patos basin (PAT) | Cristal-RS | 30^0^4'5.6"S 52º05'18.9"W |
| **M. cottoides* | MCN 17546 | Laguna dos Patos basin (PAT) | Palmares do Sul-RS | N/I |
| **M. cottoides* | MCP 23004 | Arroio Bom Jardim, Jacuí basin | Triunfo-RS | 29°50'19.0"S 51°23'25.0"W |
| **M. cottoides* | MCP 33560 | Rio Taquari, Jacuí basin | Muçum-RS | 29°10'10.0"S 51°53'06.0"W |
| *M. cottoides* | UFRGS 20254 | Rio Santa Barbara, Uruguay basin (URU) | Santo Ângelo-RS | 28º15'54.98"S 54º12'27.79"W |
| *M. cottoides* | UFRGS 20029 | Uruguay basin (URU) | Santo Antônio das Missões-RS | 28º33'09"S 55º26'14"W |
| *M. cottoides* | UFRGS 11931 | Margem BR 290, Km 10, Uruguay basin (URU) | Rosário do Sul-RS | 30^0^12´42.8”S 55^0^03´17.5”W |
| *M. cottoides* | UFRGS 14654 | Rio Negro, Uruguay basin (URU) | Rosário do Sul-RS | 31^0^28´37”S 54^0^08´19”W |
| *M. cibelae* | MZUEL 7443 | Sanga da Paca river, Mampituba basin (MAM) | Jacinto Machado-SC | 29º04'40.7"S 49º49'5.2"W |
| *M. cibelae* | MZUEL 7449 | Rio Três Forquilha, Tramandaí basin (TRA) | Vale de 3 Forquilhas-RS | 29º29'48"S 50º05'53.9"W |
| *M. cibelae* | MZUEL 7452 | Rio Maqué, Tramandaí basin (TRA) | Maquiné-RS | 29º37'2.1"S 50º15'51.8"W |
| *M. cibelae* | MZUEL 8964 | Tramandaí basin (TRA) | Maquiné-RS | 29º39'10.4"S 50º12'31.8"W |
| *M. cibelae* | LBP 14476 | Rio Maquiné, Tramandaí basin (TRA) | Maquiné-RS | 29^0^43´13.9”S 50^0^11´55.2”W |
| *M. cibelae* | MZUEL 7454 | Rio Quebrada dos Sinos, Tramandaí basin (TRA) | Caara-RS | 29º46'29.9"S 50º26'32.9"W |
| **M. cibelae* | MCP 26962 | Arroio Água Parada, (Bacia do Taquari) | Maquiné-RS | 29°39'44.0"S 50°12'44.0"W |
| **M. cibelae* | MCP 20707 | Arroio forqueta, Tramandaí basin (TRA) | Maquiné-RS | 29°36'46.0"S 50°15'44.0"W |
| **M. cibelae* | MCP 20715 | Tramandaí basin (TRA) | Maquiné-RS | 29°40'09.0"S 50°12'03.0"W |
| **M. cibelae* | MCP 29294 | Rio Três Forquilha, Tramandaí basin (TRA) | Itati-RS | 29°30'31.0"S 50°05'32.0"W |
| **M. cibelae* | MCP 20723 | Tramandaí basin (TRA) | Osório-RS | 29°57'57.0"S 50°13'45.0"W |
| **M. cibelae* | MCP 21190 | Tramandaí basin (TRA) | Osório-RS | 29°57'57.0"S 50°13'45.0"W |
| **M. cibelae* | MCP 21081 | Tramandaí basin (TRA) | Osório-RS | 29°57'57.0"S 50°13'45.0"W |
| *M. parahybae* | LBP 10741 | Macabu river, Paraíba do Sul basin | Conceição de Macabu-RJ | 22^0^04´07.8”S 41^0^54´36.2”W |
| *M. garavelloi* | LBP 10562 | Upper Paraná river basin | Upper Paraná river | 22^0^44´50.1”S 48^0^28´29.64”W |
| *M. garavelloi* | LBP 10561 | Upper Paraná river basin | Upper Paraná river | 22^0^44´50.1”S 48^0^28´29.64”W |
| *M. garavelloi* | LBP 1193 | Upper Paraná river basin | Upper Paraná river | N/I |
| *M. garavelloi* | LBP 22539 | Upper Paraná river basin | Upper Paraná river | 23^0^1´27.48”S 48^0^49´41.16”W |
| *M. garavelloi* | LBP 22540 | Upper Paraná river basin | Upper Paraná river | 23^0^1´27.48”S 48^0^49´41.16”W |
| *Microglanis* sp. | LBP 13151 | Arroio Putiá, Uruguay basin | Uruguaiana-RS | 27º11'50"S 53º42'29"W |
| *M. malabarbai* | MZUEL 17059 | Arroio Albino, Uruguay basin | São Pedro do Butiá-RS | 28º08'02.2"S 54º55'31.6"W |
| **M. malabarbai* | MZUEL 17065 | Arroio Albino, Uruguay basin | São Pedro do Butiá-RS | 28°08'02.2"S 54°55'31.6"W |
| **M. malabarbai* | MCP 37252 | arroio Alexandrino, Uruguay basin | Salvador das Missões-RS | 28°10'25.0"S 54°48'05.0"W |
| **M. malabarbai* | MCP 37187 | Arroio Pedras, Uruguay basin | 16 de Novembro-RS | 28°12'07.0"S 54°04'30.0"W |
| *M. malabarbai* | UFRGS 20253 | Riacho Araçá, Uruguay basin | Rolador-RS | 28º13'14.29"S 54º56'57.94"W |

N/I: No Information
