# Supplementary figures and images for: Reappraisal of the systematics of Microglanis cottoides (Siluriformes, Pseudopimelodidae), a catfish from southern Brazil
Source: PLoS One. 2018 Jul 5;13(7):e0199963. doi: 10.1371/journal.pone.0199963 (PMC6033443; doi:10.1371/journal.pone.0199963)

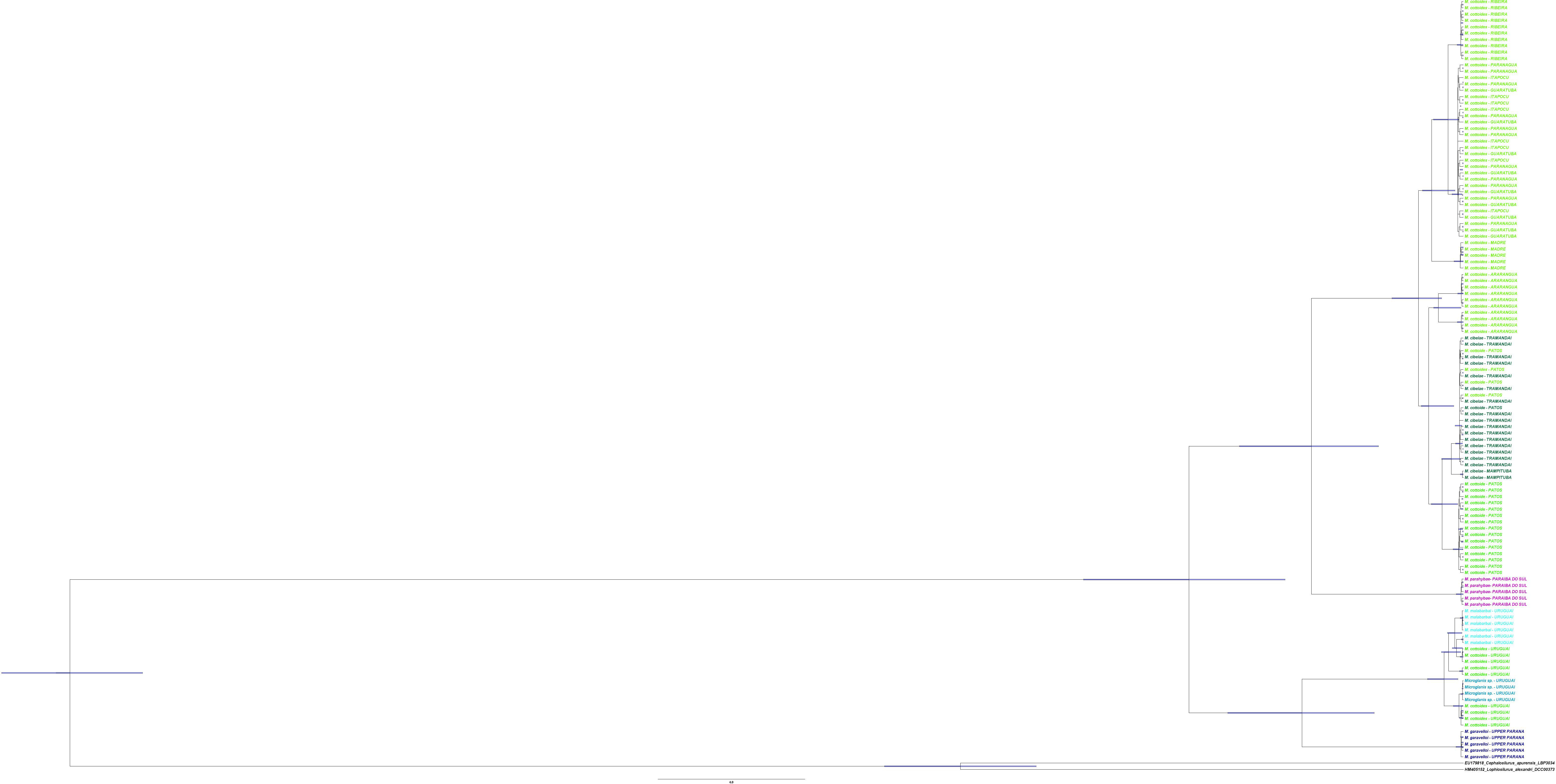

Supplement: S1 Fig — (TIF) [file pone.0199963.s002.tif]

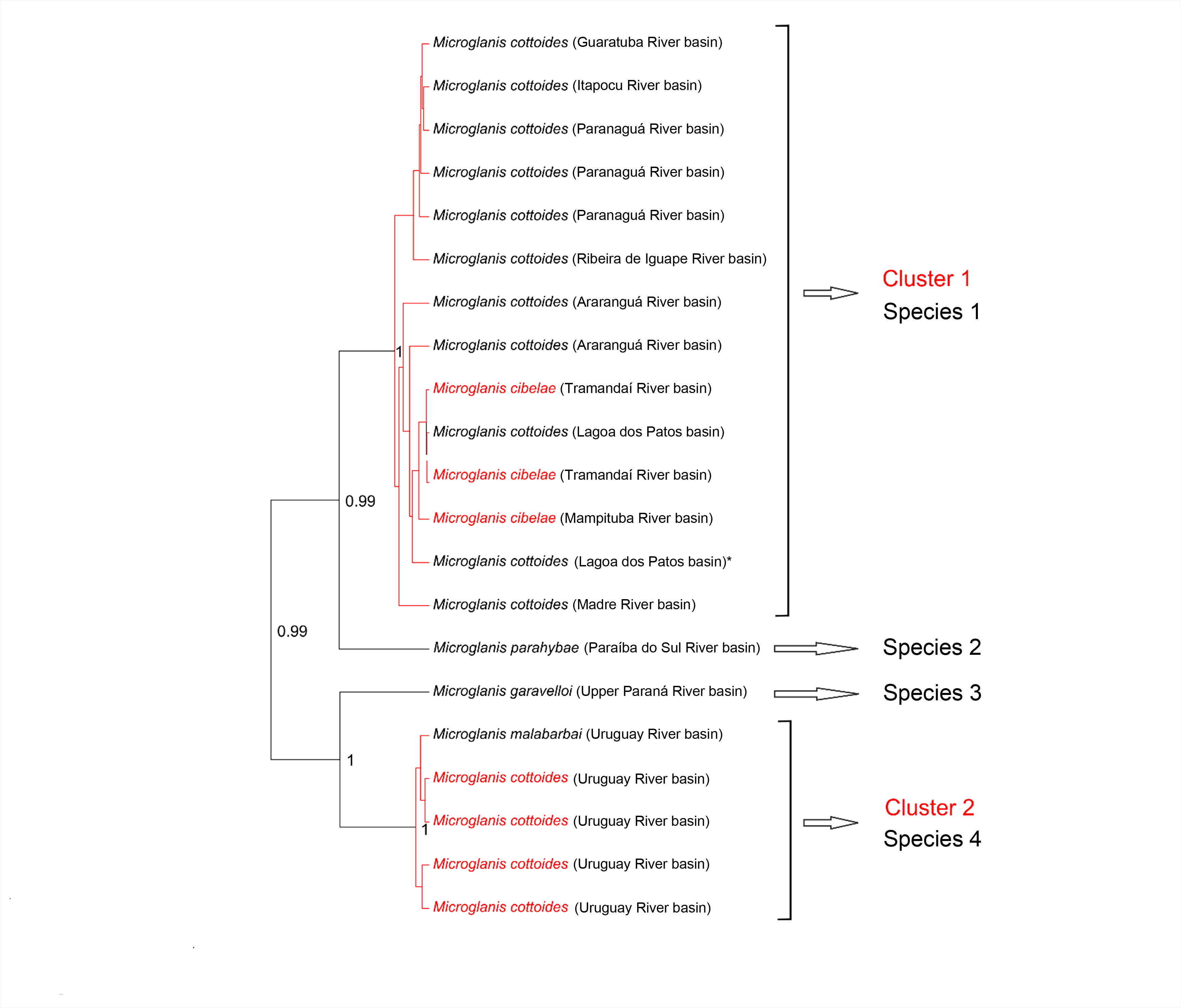

Supplement: S2 Fig — Branches highlighted in red are the result of interspecific and intra-specific branching processes of Microglanis lineages, using the GMYC model, based on the results of the phylogenetic analysis. In particular, M. cibelae associated to M. cottoides from coastal drainage and M. cottoides from the Uruguay River basin, associated with M. malabarbai. The values above the branches refer to the posterior probability. (TIF) [file pone.0199963.s003.tif]
